# Antioxidant capacities of the selenium nanoparticles stabilized by chitosan

**DOI:** 10.1186/s12951-016-0243-4

**Published:** 2017-01-05

**Authors:** Xiaona Zhai, Chunyue Zhang, Guanghua Zhao, Serge Stoll, Fazheng Ren, Xiaojing Leng

**Affiliations:** 1 Beijing Advanced Innovation Center for Food Nutrition and Human Health, Beijing Laboratory for Food Quality and Safety, Beijing Dairy Industry Innovation Team, College of Food Science & Nutritional Engineering, China Agricultural University, Beijing, 100083 China; 2 Group of Environmental Physical Chemistry, F.-A. Forel Institute, University of Geneva, Geneva, Switzerland

**Keywords:** Chitosan, Selenium nanoparticles, ROS, Lipofuscin, UV radiation, d-Galactose

## Abstract

**Backgrounds:**

Selenium (Se) as one of the essential trace elements for human plays an important role in the oxidation reduction system. But the high toxicity of Se limits its application. In this case, the element Se with zero oxidation state (Se^0^) has captured our attention because of its low toxicity and excellent bioavailability. However, Se^0^ is very unstable and easily changes into the inactive form. By now many efforts have been done to protect its stability. And this work was conducted to explore the antioxidant capacities of the stable Se^0^ nanoparticles (SeNPs) stabilized using chitosan (CS) with different molecular weights (Mws) (CS-SeNPs).

**Results:**

The different Mws CS-SeNPs could form uniform sphere particles with a size of about 103 nm after 30 days. The antioxidant tests of the DPPH, ABTS, and lipid peroxide models showed that these CS-SeNPs could scavenge free radicals at different levels. And the 1 month old SeNPs held the higher ABTS scavenging ability that the value could reach up to 87.45 ± 7.63% and 89.44 ± 5.03% of CS(l)-SeNPs and CS(h)-SeNPs, respectively. In the cell test using BABLC-3T3 or Caco-2, the production of the intracellular reactive oxygen species (ROS) could be inhibited in a Se concentration-dependent manner. The topical or oral administration of CS-SeNPs, particularly the Se nanoparticles stabilized with low molecular weight CS, CS(l)-SeNPs, and treated with a 30-day storage process, could efficiently protect glutathione peroxidase (GPx) activity and prevent the lipofusin formation induced by UV-radiation or d-galactose in mice, respectively. Such effects were more evident in viscera than in skin. The acute toxicity of CS(l)-SeNPs was tenfold lower than that of H_2_SeO_3_.

**Conclusions:**

Our work could demonstrate the CS-SeNPs hold a lower toxicity and a 30-day storage process could enhance the antioxidant capacities. All CS-SeNPs could penetrate the tissues and perform their antioxidant effects, especially the CS(l)-SeNPs in mice models. What’s more, the antioxidant capacities of CS-SeNPs were more evident in viscera than in skin.

## Background

Selenium (Se) is involved in the antioxidant defense systems of the liver and plays an important role in protecting against oxidative stress. Many studies demonstrated that Se supplementation can increase the level of enzymes such as GPx etc., prevent the accumulation of free radical species, and reduce the cellular damage [1–4]. However, the narrow margin between the effective and toxic doses limited the application of this substance [5]. The Se^0^ has thus gained more attention because of its low toxicity and excellent bioavailability compared with Se(IV) and Se(VI), since both having a strong ability to capture free radicals [6, 7]. Nevertheless, poor water solubility and the ability to easily transform into a grey analogue that is thermodynamically stable but biologically inert, makes Se^0^ difficult to be used in food and medicine fields [8, 9].

The water solubility of an insoluble substance can be greatly improved by reducing the size and increased the specific surface with convenient nanotechnology. In the past decades, nanotechnology has been used to prepare antioxidant products using minerals including silver [10], gold [11], cerium oxide [12], and platinum [13] etc., based upon their red-ox abilities. Selenium was also considered owing its multiple valence states (+6, +4, +2, 0, −1, −2) and more complex antioxidant activities [14]. In a quest to use Se^0^, many efforts have been made to design such nano-vehicles using polysaccharides, proteins, and/or lipids etc. as stabilizers [15–17]. The obtained Se nanoparticles are reported as novel compounds with excellent antioxidant properties and lower toxicity compared with other selenospecies [18]. It should be noted that in these reports the data about the effects of the stabilizers on the antioxidant functionalities of the nanosystem are still incomplete, especially on the relationships between the microstructure features and bio-activities of the whole system in vitro and in vivo.

Chitosan (CS), the N-deacetylated form of natural chitin found widely in the exoskeleton of crustaceans, insects, and fungi, has been often used as the Se^0^-stabilizer not only because of its low toxicity and bioavailability, but it can also withstand pepsin and pancreatin to a great extent [19, 20]. This naturally helps to enhance the stability of the Se^0^ system in the digestive enzyme environment. In our previous work, we compared the physicochemical properties of the Se^0^ spherical nanoparticles with a size at about 103 nm prepared through the reduction of seleninic acid with ascorbic acid in the presence of chitosan with different molecular weights [21]. We found that, although SeNPs could be stabilized using both the chitosan with low [CS(l)-SeNPs] or high molecular weight [CS(h)-SeNPs] in 30 days, the microstructure of the former seemed more compact than the latter. This divergence caused the Se release of the former more slowly than the latter in the simulated gastric, intestinal, and sweat environment. This raises a question as to whether such difference in the microstructure of SeNPs between CS(l)-SeNPs and CS(h)-SeNPs affects the bio-activities of these nanoparticles in vitro and in vivo.

As side-products of the normal metabolism, the accumulation of random molecular damage due to ROS promoted by oxidative stress is widely believed to cause cellular aging [22]. Lipofusin (LF) as the hallmark of aging is a membrane-bound cellular waste by oxidation that can be neither degraded nor ejected from the cell but can only be diluted through cell division and subsequent growth which is often found in skin and viscera [23–25]. In spite of LF formation involving complex intracellular reactions, it can be retarded by various antioxidant systems including enzymatic (e.g., GPx, SOD, etc.) and non-enzymatic antioxidant systems (e.g., vitamins E and C etc.) [26]. Many works pointed out that the level of the GPx activity could represent the state of Se uptake [3]. In addition, some reports indicated that a low status of Se was related with LF accumulation, and topical and oral Se administration of l-selenomethionine or sodium selenite could prevent LF formation induced by UV irradiation [27–29]. Therefore, the detection of GPx activity and LF levels can be used to study the antioxidant activities of CS-SeNPs.

In this work, CS-SeNPs were manufactured using chitosan with different molecular weights and with different storage times according to our previous work [21]. The inhibition of the intracellular ROS by CS-SeNPs was examined in the BABLC-3T3 and Caco-2 cell lines, designed as skin or viscera cell models, respectively. The former cell has been scientifically validated for the skin phototoxicity test [30], and the latter can represent drug intestinal absorption. The effects of CS-SeNPs on LF in skin and viscera were investigated using mice models treated with UV-radiation and d-galactose, respectively. The concerned acute toxicity of the nanoparticles was also verified.

## Methods

### Reagents

The seleninic acid (H_2_SeO_3_), 2,2′-Azino-bis(3-ethylbenzothiazoline-6-sulfonic acid (ABTS), 2,2-Diphenyl-1-picrylhydrazyl (DPPH), d-(+)-galactose, reduced l-glutathione (buffered aqueous solution, ≥10 units/mg protein, recombinant, expressed in *E. coli*), 2,3-Diaminonaphthalene (DAN), 2′,7′-Dichlorofluorescein diacetate (DCFH-DA), 2,4,6-Tris(dimethylaminomethyl) phenol(DMP-30), and glutaraldehyde were purchased from Sigma Aldrich, Inc. (St. Louis, MO, USA). Dulbecco’s Modified Eagle Medium (DMEM), fetal bovine serum, Penicillin-Streptomycin Solution (100×), GlutaMAXTM-1 (100×), MEM Nonessential Amino Acid Solution (NEAA, 100×), dimethyl sulfoxide (DMSO), potassium phosphate (PBS, pH 7.4), Trypsin–EDTA, formalin, hematoxylin, and eosin were purchased from Solarbio Science & Technology Co., Ltd. (Beijing, China). The chitosan with a molecular weight of less than 3 kDa (CS3) and 200 kDa (CS200) (Poly-β-(1,4)-d-glucosamine, DD > 85%) were purchased from Jinan Haidebei Co., Ltd. (Shandong, China). The other regents included acetic acid, ascorbic acid, HClO_4_, HNO_3_, HCl, H_2_SO_4_, EDTA, ethanol, methanol, acetone, cyclohexane, potassium persulfate (K_2_S_2_O_8_), Na_2_HPO_4_·12H_2_O, NaH_2_PO_4_·2H_2_O, egg lecithin, FeSO_4_, trichloroacetic acid (TCA), 2-Thiobarbituric acid (TBA), NaCl, hydroxylamine hydrochloride, cresol red, quinine sulphate, ammonium hydroxide, stearic acid, white petrolatum, propylene glycol, triethanolamine, and edetate disodium dehydrate were of analytical grade. The edible oil, wax, and rosin were from the local market.

### Preparation and characterization of CS-SeNPs

CS-SeNPs were manufactured according to the method described in our previous work [21]. These nanoparticles stabilized with CS3 and CS200 were denoted as CS(l)-SeNPs and CS(h)-SeNPs, respectively. The numbers 0 and 30 in CS(l)-SeNPs-0 day, CS(l)-SeNPs-30 days, CS(h)-SeNPs-0 day, and CS(h)-SeNPs-30 days were used to distinguish the nanoparticles manufactured immediately and those followed by 30-days storage, respectively. The Se concentration of all of the CS-SeNPs stock was adjusted to 0.1 mol/L.

The morphology of these nanoparticles was observed by means of scanning transmission electron microscopy (STEM). The sample solution was dropped on a carbon-coated copper grid for 5 min and the excess solution was removed and dried in the air for 30 min. The observations were performed using a Hitachi S-5500 STEM (Hitachi High Technologies America, Inc. IL, USA) with an operation voltage of 30 kV. The images were acquired using a Gatan high-angle annular bright field scintillating detector. The hydrodynamic size and zeta potential of the nanoparticles were measured using a Delsa–Nano Particle Analyzer (A53878, Beckman Coulter, Inc., CA, USA).

### Assay for antioxidant activities of CS-SeNPs in vitro

The antioxidant abilities of the CS-SeNPs samples were presented as the radicals scavenging activity (RSC%) in DPPH, ABTS, or lipid peroxide. The value of RSC% was calculated using the following formula:

1 $${\text{RSC}} \, ( \%) = \frac{{{\text{A}}_{ 0} - {\text{A}}_{1} }}{{{\text{A}}_{ 0} }} \times 100\%$$

where A_0_ is the absorbance of the control and A_1_ is the absorbance of the mixed solution of the antioxidant and free radical agent.

The RSC% in DPPH was determined according to the method described in the work of Xu [31]. A 0.2 mL dose of the nanoparticle sample was mixed vigorously with 3.8 mL of DPPH radical ethanol solution (final DPPH concentration: 0.1 mmol/L), and then kept at room temperature in the dark for 30 min. The absorbance was measured at 517 nm with a UV spectrophotometer (UVmini-1240; Shimadzu, Japan).

The RSC% in ABTS was determined according to the work of Re [32]. The stock was prepared by mixing 0.5 mL of 14 mmol/L ABTS and 0.5 mL of 4.9 mmol/L K_2_S_2_O_8_, and then keeping them in the dark at room temperature for at least 12 h in a 1.5 mL tube. The absorbance of the ABTS solution was adjusted by PBS buffer (pH 7.4, 150 mmol/L) to 0.70 ± 0.02 at 734 nm. The measurement was performed at 734 nm exactly 4 min after mixing 900 μL of the diluted ABTS solution with 100 μL of the nanoparticle sample.

A modified TBA-reactive species assay was used to measure the formed lipid peroxide with egg yolk lecithin homogenates as a lipid-rich media [33]. The occurrence of malondialdehyde (MDA), a secondary end product of the oxidation of polyunsaturated fatty acids, was used as an index of lipid peroxidation. The MDA reacted with TBA to yield a pinkish-red chromogen with an absorbance maximum at 532 nm. One gram of egg lecithin was sonicated in 50 mL PBS buffer (pH 7.4, 150 mmol/L) at 4 °C for 30 min. After mixing 0.5 mL of this solution with 0.1 mL of the nanoparticle sample, the total volume was made up to 1 mL with distilled water. The obtained mixture was added into 0.05 mL of FeSO_4_ (70 mmol/L) and then incubated at 37 °C for 30 min. We added 0.5 mL of TCA (10%, w/w) into the above incubated solution, followed by 0.5 mL of TBA (1%, w/w). The final mixture was vortexed and heated in a boiling water bath for 60 min. After cooling, the solution was centrifuged at 3000×*g* for 10 min. The upper organic layer was collected and measured at 532 nm.

### Cell lines and culture

Two types of cell lines, purchased from China Infrastructure of Cell Line Recourses (Beijing, China), were used in this work. One was the mouse embryonic fibroblast BABLC 3T3 cells cultured in DMEM media supplemented with 10% (v/v) bovine calf serum and 1% (v/v) GlutaMAX, and the other was Caco-2 cells cultured in DMEM containing 10% (v/v) bovine calf serum and 1% (v/v) NEAA. Both cell lines were incubated at 37 °C in a humidified incubator with 5% CO_2_.

### Cell viability assay

The MTT assay was used to determine the cytotoxicity of the CS-NPs [18] and a MTT [3-(4, 5-dimethylthiazol-2-yl)-2, 5-diphenyltetrazolium bromide] cell viability/cytotoxicity assay kit (Beyotime Biotechnology, Jiangsu, China) was used to determine cell viability. Healthy cells can reduce the MTT to a purple formazan dye. Both cells were seeded in a 96-well microplate with 5 × 10^3^ cell/well and 0.1 mL growth medium/well for 24 h, respectively. After that, each cell line was treated by incubating with CS(l)-SeNPs, CS(h)-SeNPs, and H_2_SeO_3_, respectively. The Se concentrations varied between 50 and 500 μmol/L. The incubation was performed for another 24 h. The control groups were left untreated. The absorbance was measured at 570 nm with a Thermo Fisher Scientific Varioskan^®^ Flash Multimode Reader (Thermo Scientific, USA); the viability was determined based on the manufacturer’s instructions.

### Measurement of the intracellular ROS generation

The intracellular ROS accumulation was evaluated using a DCF fluorescence assay [34]. The BABLC-3T3 and Caco-2 cells were seeded in a 96-well microplate with 9 × 10^4^ cell/well and 0.1 mL of growth medium/well for 24 h, respectively. After that, the growth medium was removed and the wells were washed with the PBS buffer (pH 7.4, 10 mmol/L). The cells were then incubated with CS(l)-SeNPs, CS(h)-SeNPs, and H_2_SeO_3_, respectively. The Se concentrations varied between 50 and 500 μmol/L. The control groups were treated without the above Se samples. The incubation was performed for another 24 h. At the end of the incubation, the cells were rinsed three times with a cold PBS buffer (4 °C) in order to remove the excess nanoparticles around the cells. Finally, these cells were incubated with DCFH-DA at a final concentration of 20 μm at 37 °C for 60 min. The level of the intracellular ROS was examined by detecting the fluorescence intensity conducted with a Thermo Scientific Varioskan^®^ Flash Multimode Reader (with the excitation and emission wavelength set at 488 and 525 nm, respectively).

### Animals and treatments

The Kunming (KM) mice (Strain code: 202, initial weight: 20 g to 25 g) were purchased from Vital River Laboratories Co., Ltd. (Beijing, China). These mice were allowed free access to food and water. All animal procedures were conducted in accordance with the Animal Care and Use Guidelines of the China Council on Animal Care (Regulations on the Administration of Laboratory Animals, 2013 Revision published by the State Council on July 18, 2013). The protocol complied with the guidelines of China Agriculture University for the care and use of laboratory animals.

### Acute toxicity

A total of 120 KM mice were randomly divided into 12 groups, with equal numbers of female and male in each group. The CS(l)-SeNPs and H_2_SeO_3_ were administered by single intragastric administration with increasing doses (1.43-fold), and the mortalities were recorded within 14 days. The values of LD50 and 95% confidence were calculated by Trimmed Spearman-Kaber’s Method [35].

### Transdermal tests of CS-SeNPs

**Fig. 1:**
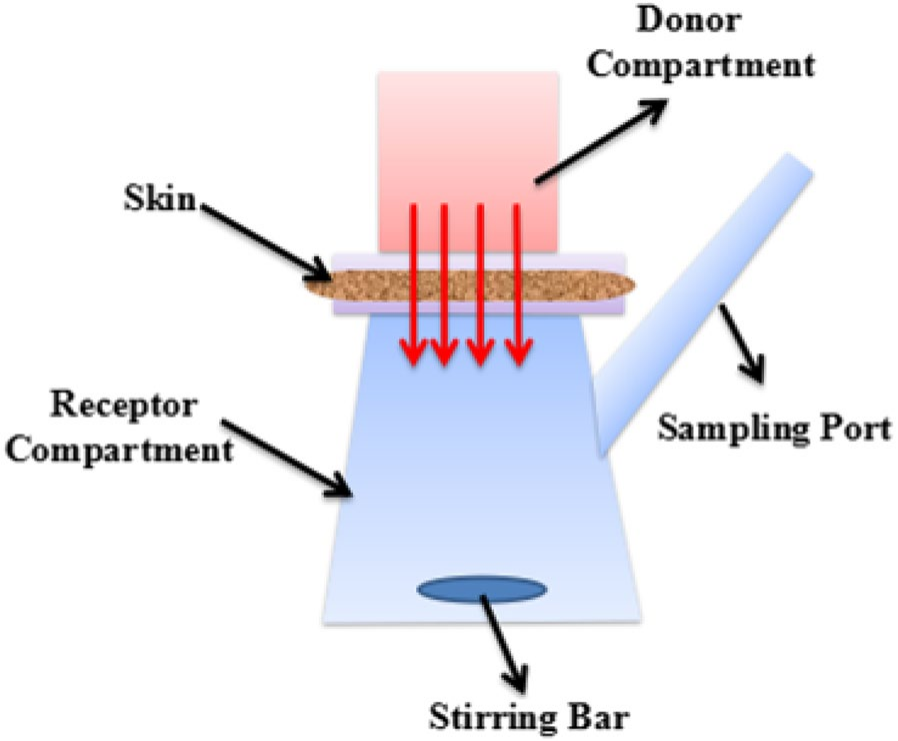
The schematic diagram of the Franz diffusion cell system

The transdermal tests were conducted using a vertical Franz diffusion cell system (TP-6, Tianguang Photoelectric Instrument Co., Tianjin, China) equipped with 6 identical diffusion cells. Each cell contained a donor compartment and a receptor compartment filled with 17 mL normal saline. These two compartments were connected through a circular channel with a cross-sectional area of 3.4 cm^2^ (Fig. 1). A piece of mouse dorsal skin, free of subcutaneous fat, tissues, blood vessels, and epidermal hairs, was mounted on the channel as a diffusion membrane with the stratum corneum facing the donor compartment. The sample solutions were added in the donor compartment for 6 h, and the substance through the skin was collected with the normal saline stirred at a rate of 600 rpm at 37 °C.

The Se concentration in the donor compartment was kept at 2 mM, and the Se through the skin was collected and determined by means of hydride-generation atomic fluorescence spectrometry (AFS-230E, Beijing Haiguang Instrument Co., Beijing, China) as the following procedure noted in literature [36]: the collected solutions were filtered with 0.45 μm Millipore filter and then heated with 5 mL HClO_4_/HNO_3_ (1/3, V/V) mixture and 3 mL HCl to eliminate the organic impurities. After cooling, 5 mL deionized water, 1 mL EDTA (1%), 1 mL hydroxylamine hydrochloride (10%), and 0.2 mL cresol red (0.02%) was added successively into the filtrates. The pH was adjusted to 1.5 with HCl or NH_4_OH. The solutions were incubated at 60 °C for 30 min after adding 1 mL DAN (0.1%), and then 5 mL cyclohexane was added into the cooled solutions by shaking. After standing for 30 min, the supernatant was collected and then measured by AFS with excitation and emission wavelengths at 376 and 520 nm, respectively.

### Bioactivity of CS-SeNPs in the UV-induced skin damage model

A total of 48 male mice were randomly divided into 6 groups with 8 mice in each group. More details of the procedure were noted in Table 1. The dorsal skin of the mice was denuded with a wax/rosin mixture (1:1, w/w) every 10 days [37]. The drug vehicle was prepared using a standard low-Sun Protection Factor (SPF) cosmetic base formula [38]. The samples were stirred to smooth pastes with the vehicle. The paste was used 30 min before the UV treatment. The irradiation was made using a UV lamp (TL12rs 40 W UVB lamp, Philips, Poland) at a dose of 1.0 kJ/m^2^ and lasted for 15 days [39]. Then the mice were sacrificed and the dorsal skins were carefully removed and collected to determine LF content and GPx activity. The pathological study of skins was also performed.

**Table 1:** Group, drug dose and UV treatment parameters for the topical tests of the CS-SeNPs

Group	Se sample	Dose [mg/kg body weight]	UV radiation
UV-induced skin damage			
1	–	/	–
2	Drug vehicle	/	+
3	CS(l)-SeNPs (30 days)	1	+
4	CS(l)-SeNPs (30 days)	10	+
5	CS(h)-SeNPs (30 days)	1	+
6	H_2_SeO_3_	1	+

### Bioactivity of CS-SeNPs in the d-galactose induced mouse aging model

A total of 48 male mice were randomly divided into 6 groups with 8 mice in each group. More details of the procedure were noted in Table 2. Along with the oral supplementation of the tested samples, a dose of 200 mg/kg d-galactose (drug/body weight) per day was intraperitoneally injected for 4 weeks. The normal saline was used as the blank. Then the mice were sacrificed and the livers and kidneys were immediately collected to determine the LF content and GPx activity.

**Table 2:** Group, drug dose, and d-galactose parameters for the topical tests of CS-SeNPs

Group	Se sample	Dose [mg/kg body weight]	d-Galactose
d-Galactose induced aging			
1	–	/	–
2	Drug vehicle	/	+
3	CS(l)-SeNPs (30 days)	1	+
4	CS(l)-SeNPs (30 days)	10	+
5	CS(h)-SeNPs (30 days)	1	+
6	H_2_SeO_3_	1	+

### LF and GPx assessment

LF content was determined by a modified fluorescence method described in the work of Harvey et al. [40]. A saline solution containing of 10% (w/w) skin or viscera was freshly homogenized in an ice-water bath. After mixing 2 mL of this homogenate with a 4 mL of the CHCl_3_/MeOH (2:1, v/v) extraction agent, the solution was sonicated for 30 min and then centrifuged at 5000 rpm for 1 min. The lower chloroform phase in the tube was carefully collected with a syringe for the following measurement. The LF content was determined using the following relationship:

2 $${\text{Lipofuscin content }}\left( {\text{mg/g tissue}} \right)\, = \,\frac{{{\text{I }}_{\text{sample}} - {\text{I}}_{\text{control}} }}{{{\text{I}}_{\text{standard}} }} \times \,{\text{C}}_{\text{standard }} \left( {0.1\, {\text{mg}}/{\text{mL}}} \right)\, \times \,\frac{{ {\text{V}}_{\text{extract }} \left( {4\,{\text{mL}}} \right)}}{{{\text{W}}_{\text{tissue}} \,\left( {\text{g}} \right)}}$$

where I_sample_ is LF, I_control_ is the CHCl_3_/MeOH extraction agent, and I_standard_ is the calibration against a quinine sulfate solution (1 µg/mL, 0.1 mol/L H_2_SO_4_). The wavelengths of the excitation and emission were 365 and 435 nm, respectively.

The GPx activity, expressed as NU/mg protein, was determined using a Total Glutathione Peroxidase Assay Kit according to the manufacturer’s protocol (Beyotime Biotechnology, Jiangsu, China). The protein concentrations were determined by means of Bradford dye-binding assay using bovine serum albumin as the standard [41].

### Histological measurements and ultrathin sections for SEM

The histological tests of dorsal skin from the mice used for the UV-radiation test were performed in accordance with standard laboratory procedures. The biopsy skin samples (2 cm × 3 cm) were cut into small pieces, fixed in 10% formalin, and then embedded in paraffin. The samples were sliced into 2-µm-thick sections and then stained with hematoxylin and eosin staining. The observations were performed using an optical microscope controlled with TSView software in version 7.0 (Chong Qing Optical and Electrical Instrument Co., Ltd. Chongqing, China).

KM mice were deprived of food for over 24 h and were orally administered the CS-SeNPs solution and the CS-SeNPs lotion at a dosage of 25 mg Se/kg mice on the skin. After 6 h of exposure, biopsy samples from the small intestines and dorsal skin were immediately obtained for SEM observation. The ultrathin sections were made as following [42]. The small intestines and dorsal skin were quickly sliced into small pieces (1 mm × 1 mm), and then washed and fixed with 2.5% glutaraldehyde in PBS buffer (pH 7.4, 10 mmol/L). The fixed samples were dehydrated with graded ethanol solutions (70, 80, 90, and 100%, v/v, ethanol/water) and 50% acetone (v/v, acetone/ethanol), and then dehydrated twice with pure acetone. Each dehydration process lasted for 15 min. These samples were embedded in graded QUETOL 651 resin solutions (1/3, 1/1, 3/1, v/v, resin/acetone) and pure resin (with DMP-30) overnight. After standing for 24 h at 60 °C, the samples were cut into ultrathin pieces of about 70-nm thickness with a Leica EMUC6 ultramicrotome and then placed on a carbon-coated copper grid. Digital images were acquired with a Zeiss Merlin Scanning Electric Microscope (Germany) and elementary analysis was conducted with a Horiba INCA 450 energy dispersive x-ray analysis spectroscopy.

### Statistical analysis

All experiments were conducted in triplicate and expressed as mean ± SD. Statistical analysis was performed using Origin 8.5 and SPSS 16.0. The comparison was performed with χ^2^ or one-way ANOVA, followed by Dunnett’s multiple comparison tests. Statistically significant differences between groups were defined as *p* < 0.05.

## Results and discussion

### Characterization of chitosan stabilized selenium nanoparticles

**Fig. 2:**
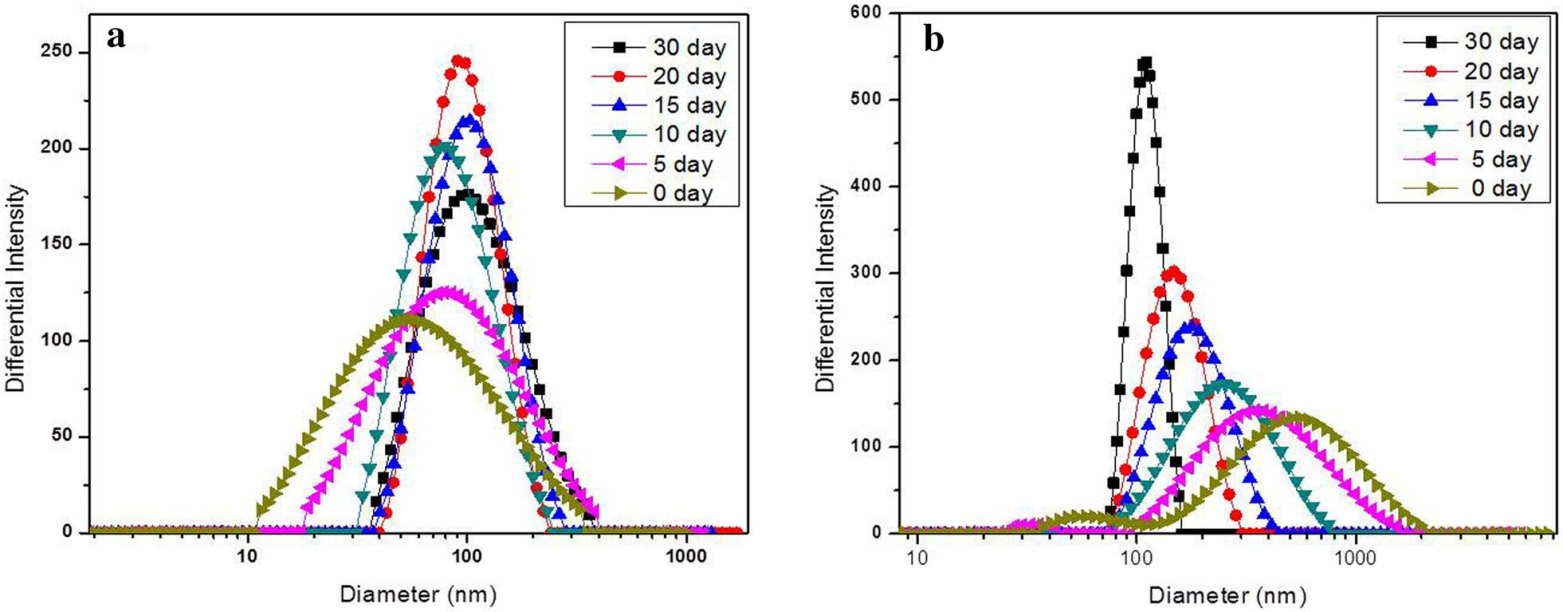
The size parameters of CS-NPs. (**a**) CS(l)-SeNPs; (**b**) CS(h)-SeNPs

The preparation of the CS-SeNPs was performed according to our previous work [21]. The CS(l)-SeNPs (Fig. 2a) formed uniform small sphere particles with a size of about 50 nm, while the CS(h)-SeNPs (Fig. 2b) formed loose and irregular aggregates with an average size of more than 350 nm caused by the bridging effect of the long macromolecular chains during the initial stage of their formation. In 30 days, via a “bottom-up” growth or “top-down” shrinkage process, respectively, both the size of the CS(l)-SeNPs and CS(h)-SeNPs tended to be about 103 nm. As depicted in the previous work, the cores of CS(h)-SeNPs were more scattered than those of CS(l)-SeNPs. The zeta potential of CS(l)-SeNPs decreased from 49.5 ± 0.9 to 33.5 ± 1.0 mv and that of CS(h)-SeNPs changed from 65.9 ± 0.1 to 44.8 ± 0.6 mv. The comparison of the zeta potential values indicated that the loose microstructure of CS(h)-SeNPs was caused by its relatively high intermolecular electrostatic repulsion and bridging effects. It was believed that such a loose microstructure of CS(h)-SeNPs led to its relatively higher Se release rate compared with that of CS(l)-SeNPs [21].

### Antioxidant capacities of CS-SeNPs in vitro

**Fig. 3:**
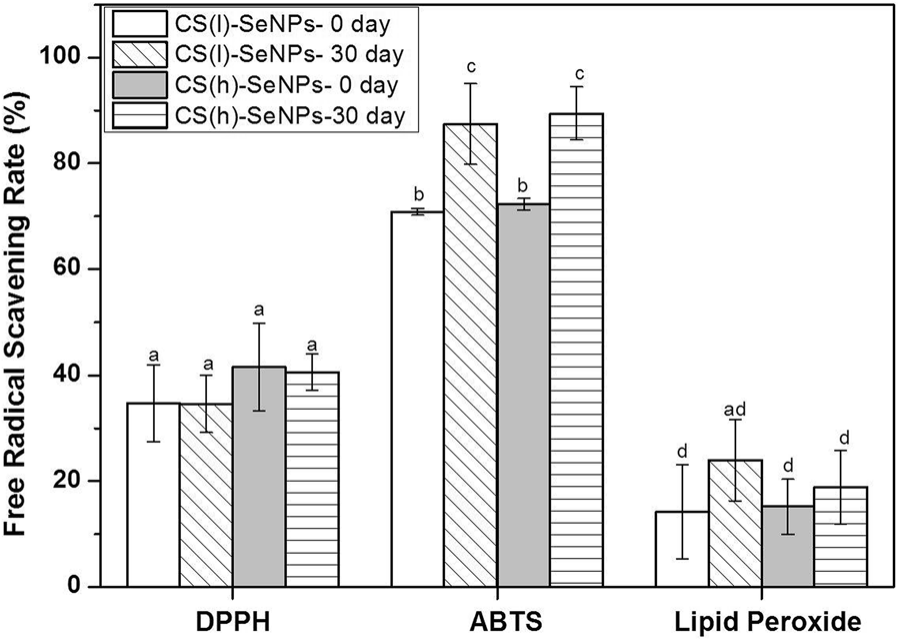
Comparison of the antioxdiant capacities of CS-SeNPs in DPPH, ABTS and lipid peroxidation systems. The *different letter* markers denote the significant mean difference at *p* < 0.05

The antioxidant capacities in vitro of the CS-SeNPs were investigated using the assays reported in literature, most of them can be classified into two types [44]: assays based on electron transfer (ET-based) such as DPPH and ABTS, and assays based on hydrogen atom transfer (HAT-based) reactions such as lipid peroxidation, depending upon the chemical reactions involved. Among them, DPPH and lipid peroxidation were carried out in hydrophobic media, while ABTS was in hydrophilic media. In order to understand the multifaceted aspects of the CS-SeNPs, the above assays were all used and results were compared in Fig. 3.

It was observed that the values of RSC% in ABTS were higher than those in DPPH and lipid peroxidation. This feature was due to the effect of the high water solubility of the nanoparticles, which led to the separation of the Se nanoparticle-rich water phase from the free radical-rich lipid phase, and thus reduced the ability of Se^0^ to capture the free radicals. The somewhat higher values of RSC% in DPPH than in lipid peroxidation indicated that the nanoparticles were more likely to ET-based reaction rather than HAT-based reaction. This behavior was different from that of some organic antioxidants such as rutin, which could react quickly with lipid peroxyl radicals but not nitrogen radicals [42]. The discussion about the difference between Se and organic antioxidants was not discussed because it was beyond the scope of this work.

Figure 3 also revealed the storage effect on the antioxidant capacities of CS-SeNPs. It was observed that the RSC% of the nanoparticles was enhanced by approximately 25% after a treatment of 30 days storage in ABTS assay. Such enhancement was normally caused by the protection of the stabilized CS shell on the antioxidant activity of Se during storage. This effect was not significant in DPPH and lipid peroxidation (*p* < 0.05), which was probably due to the low level of RSC% concealing the difference between these assays. Anyway, the use of the stabilized nanoparticles was the best choice for the following experiments. No effect of CS molecular weight was observed in all tests in vitro. Although the CS molecular weight could affect the Se release rate via the modification of the nano-carrier microstructure, the minor difference of the released Se quantities was not serious enough to disturb the antioxidant capacities in the present experimental conditions.

### ROS inhibition effects and cytotoxicity of CS-SeNPs in vitro

**Fig. 4:**
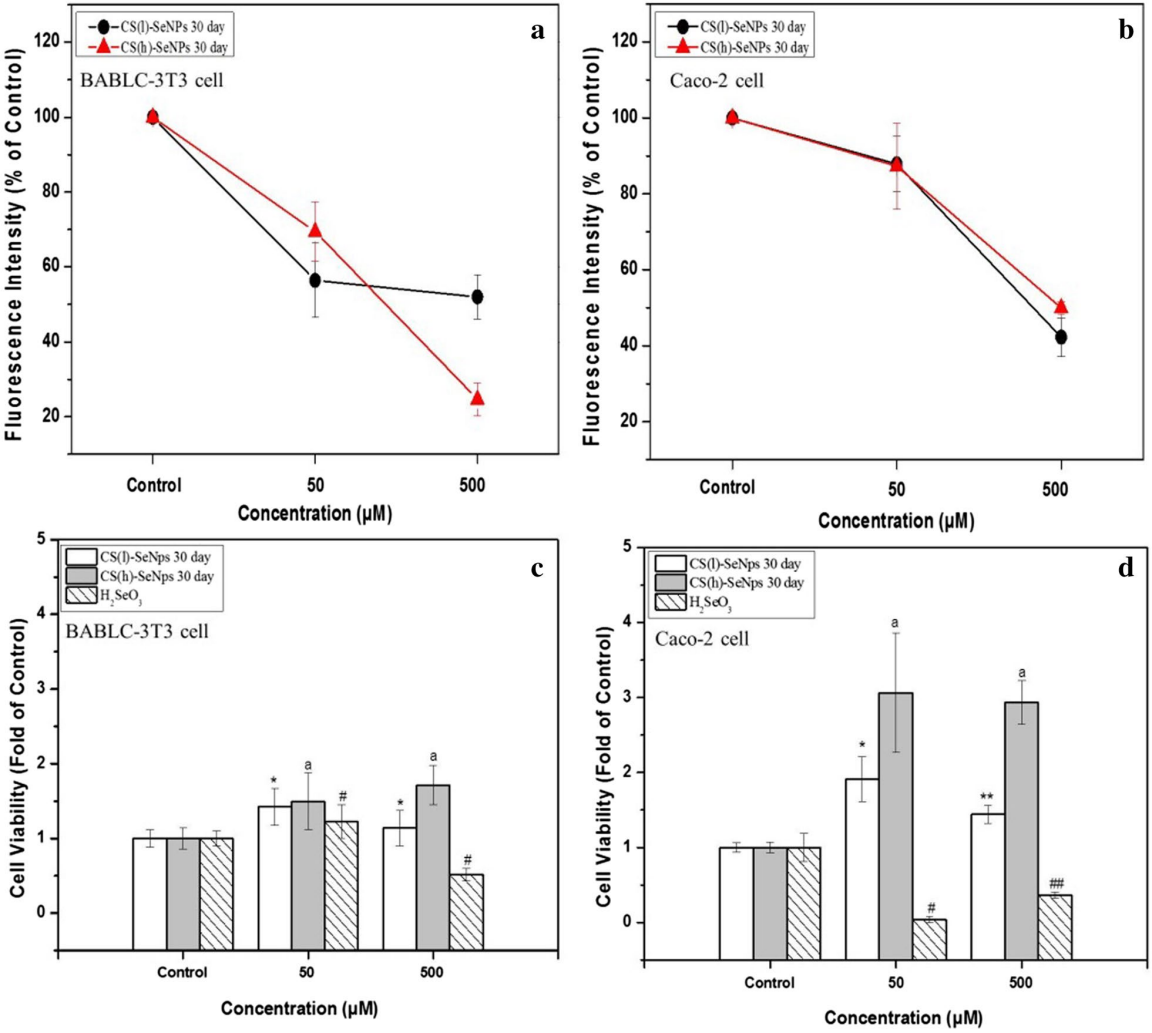
Comparison of the CS-SeNPs effects on ROS accumulations and cell viabilities in cell lines. **a**, **c** BABLC-3T3 cell, **b**, **d** Caco-2 cell lines. The *different markers* denote the significant mean difference at *p* < 0.05

An appropriate antioxidant capacity of Se nanoparticles can be used to inhibit ROS accumulation in cell, while an excessive one will result in cytotoxicity. Since the skin and digestive tract were targeted in this work, the BABLC 3T3 (a) and Caco-2 (b) cell lines were used as skin [30] and intestinal cell [45] models to test ROS inhibition effects and cytotoxicity of CS-SeNPs (Fig. 4).

Both CS(l)-SeNPs and CS(h)-SeNPs could inhibit ROS accumulation in BABLC-3T3 cell lines in a dose-dependent manner, but a larger rate of divergence existed, and an abnormal strong inhibition effect was observed with a high dose (500 μM) of CS(h)-SeNPs. While in Caco-2 cell lines, the inhibitory effects of both nanoparticles increased at almost the same rate as the drug dose.

The Se nanoparticles were cytotoxic. Zheng et al. have studied the properties of a grey Se stabilized with polyethylene glycol, PEG-SeNPs, in HepG2 cell lines [43]. They found that the PEG and Se had a synergetic effect on cell apoptosis via the induction of mitochondrial dysfunction. However, compared with H_2_SeO_3_, it was obviously observed that CS nano-systems could effectively reduce the selenium cytotoxicity in BABLC-3T3 (Fig. 4c) and Caco-2 (Fig. 4d) cell lines, respectively. The values of the cell viability of CS(h)-SeNPs were generally higher than those of CS(l)-SeNPs. Nevertheless a dose-dependent manner could be observed for CS(l)-SeNPs, but not for CS(h)-SeNPs. The properties of the former needs to be further studied, where the relationship between the physicochemical properties of the nanoparticles and biochemical properties of the cells should be considered. In this work, the CS(l)-SeNPs were preferentially used in the subsequent tests.

### Penetration tests of CS-SeNPs

The bio-activities of nanoparticles are related to their penetration ability. This ability can be affected by the nanoparticle surface coatings and also the biochemical characteristics of target organelle or tissue [46]. Since the skin contact and intestinal intake were concerned in this work, the dorsal skin and intestinal tissues of mice were used as models to test the transdermal capacities of the CS-SeNPs, respectively.

**Fig. 5:**
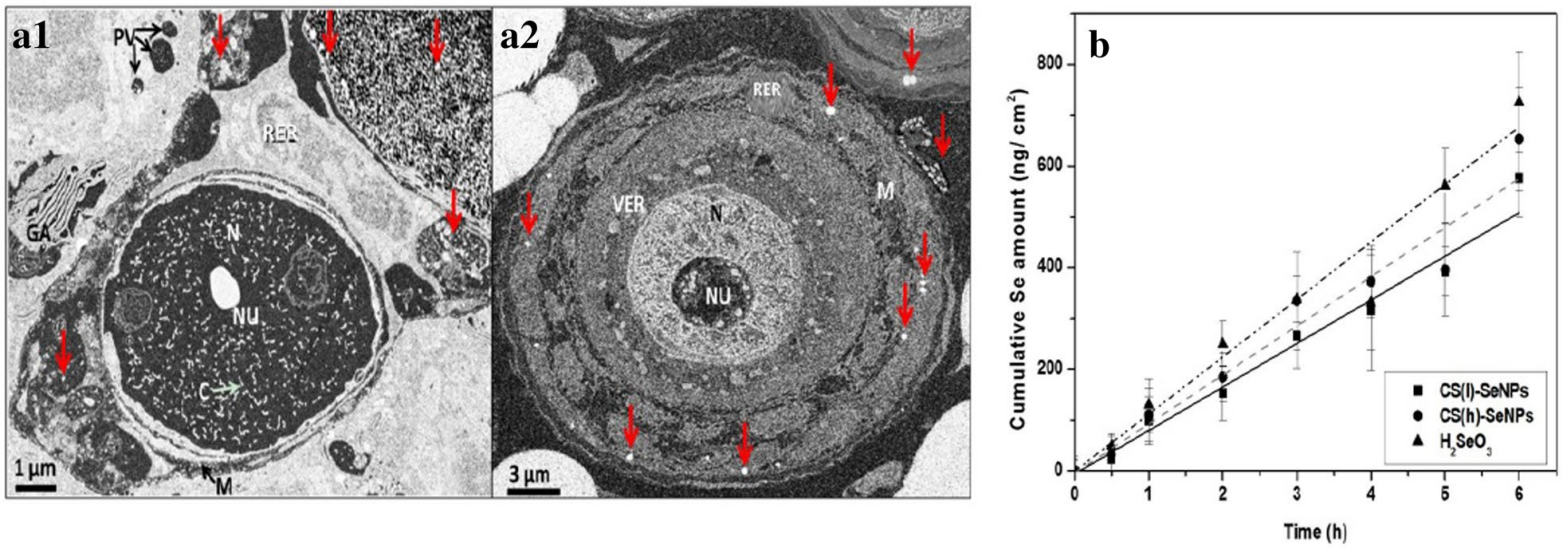
SEM observation and transdermal tests of CS-SeNPs in mice tissue. **a** Small intestine (**a1**) and skin (**a2**). *N* nucleus, *NU* nucleolus, *C* chromatin, *RER* rough endoplasmic reticulum, *M* mitochondria, *VER* vacuolization of the endoplasmic reticulum, *GA* Golgi apparatus, and *PV* pinocytic vesicle. The Se particles in the cell are marked with *red arrows*. **b** Comparison of the transdermal kinetics of the CS-SeNP and H_2_SeO_3_

Figure 5 exhibited SEM observation of Se nanoparticles in the dorsal skin and intestinal tissues of mice (a) and compared the transdermal kinetic data of CS(l)-SeNPs, CS(h)-SeNPs, and H_2_SeO_3_ (b) in the skin. The kinetic analysis of the intestinal system was not performed, because the Se quantity before the penetration process could be affected by the portion of Se released in intestine tract [21], and the state of Se accumulation on the surface of intestine wall should be also considered. As shown in Fig. 5a, it was observed that Se nanoparticles could easily penetrate cell membrane and stay near the rough endoplasmic reticulum and mitochondria. In contrast, recent reports have shown the interactions of Se nanoparticles with mitochondria [47] and lysosome [48]. Obviously, it appears that the distribution of nanoparticles in cell was quite broad.

Passive diffusion was denoted as the principle penetration mode of SeO_3_^2−^ [49] while endocytosis often happened in nanosystems [14]. Nevertheless, the transdermal Se amount of CS(l)-SeNPs, CS(h)-SeNPs, and H_2_SeO_3_ was very close and approximately showed a linear relationship with time. The rate values were at about 85.7 ± 5.5, 95.7 ± 3.5 and 112.9 ± 0.8 ng/cm^2^h, respectively (Fig. 5b), indicating that Se delivery efficiency of the CS-SeNPs was considerable to that of selenite ion diffusion.

### Antioxidant capacities of CS-SeNPs in vivo

**Fig. 6:**
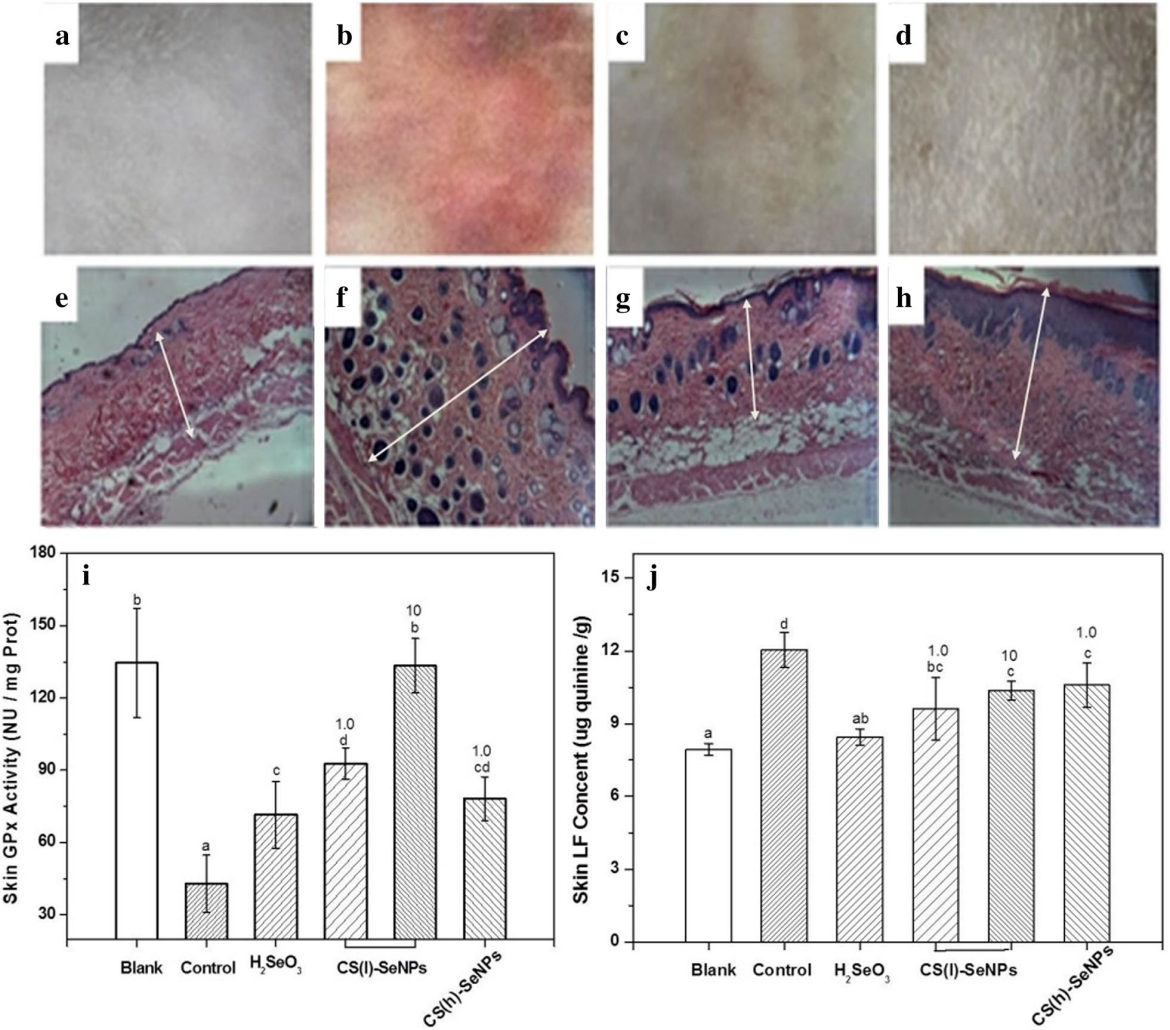
Optical micrographs of the mice skin and comparison of the GPx (i) and LF levels (j) after UV-radiation. **a**–**d** skin surface; **e**–**h** skin cross section. **a**, **e** unirradiated group; **b**, **f** UV-irradiated and treated with blank lotion; **c**, **g** UV-irradiated and treated with the CS-SeNPs; and **d**, **h** UV-irradiated and treated with H_2_SeO_3_. H&E stain, 400× magnification, the thickness of the granular layers is marked with the *double-headed arrows*. The *different letter* markers denote the significant mean difference at *p* < 0.05

**Fig. 7:**
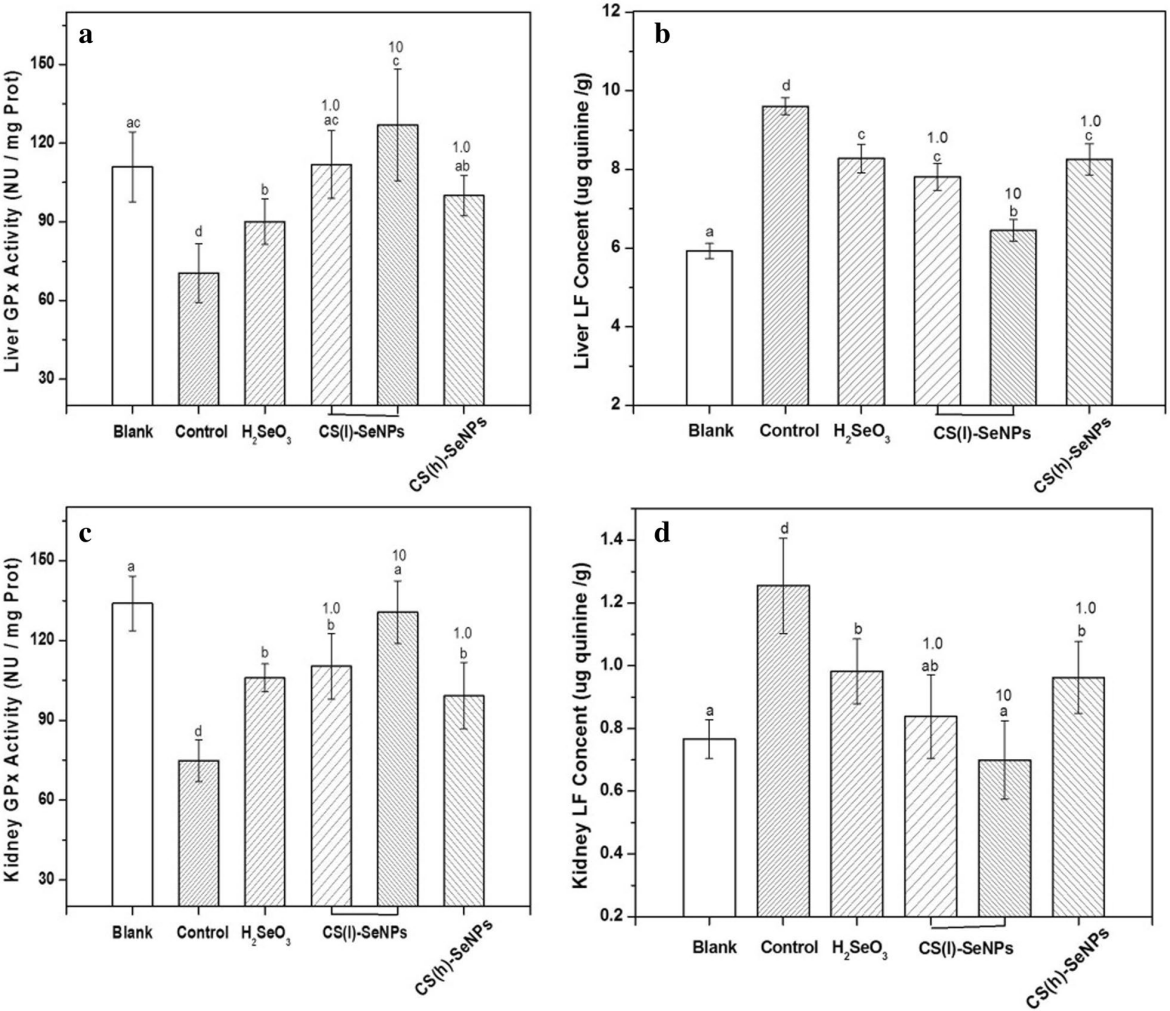
Comparison of the bio-activities of the orally administered CS-SeNPs on the livers or kidneys of the mice. **a**, **b** GPx, LF levels in the livers; **c**, **d** GPx, LF levels in the kidneys. The *different letter* markers denote the significant mean difference at *p* < 0.05

The investigations of the CS-SeNPs antioxidant capacities in vivo were conducted using the KM mouse skin (Fig. 6) or viscera (Fig. 7) treated with UV-radiation or d-galactose, respectively.

### UV-radiation system

Figure 6 exhibited the optical micrographs of the mouse skin (a, b, c, and d: skin surfaces; e, f, g, and h: skin cross-sections) and compared the GPx (i) activity and LF level (j) after UV-radiation. The surface (Fig. 6a) and cross-section (Fig. 6e) of the unirradiated skin were used as the blank, which had relatively high GPx activity and low LF. After a 15-day UV-radiation treatment, a palpable pathological pigmentation could be observed on the surface (Fig. 6b), and a number of dark granules appeared in the cross section (Fig. 6f). Meanwhile, the level of GPx activity was sharply reduced and LF increased. This irradiated group, without any antioxidant treatment, was used as the control. Under the same dose of UV radiation, the level of the pathological pigmentation and the thickness of the granular layer were greatly reduced for the skin treated with CS-SeNPs (Fig. 6c, g) or H_2_SeO_3_ (Fig. 6d, h), respectively. For these two Se samples, the former was better to protect GPx activity than the latter in respect to the same Se dose (1 mg/kg, drug/body weight). As for the two types of CS-SeNPs, CS(l)-SeNPs was better than CS(h)-SeNPs, and the concerned effect could be improved in a dose-dependent manner. The LF level could be reduced by CS-SeNPs. However, no significant dose-dependence was observed in the present dose range.

### d-galactose system

Figure 7 compares the effects of CS-SeNPs on GPx activity and LF level in KM mouse livers and kidneys treated with d-galactose, respectively. In both viscera models, d-galactose increased the LF level and reduced GPx activities. Well, the presence of the Se substance could weaken the effects of d-galactose. Similar results were reported elsewhere [50]. Among these Se substances, CS(l)-SeNPs was better than both H_2_SeO_3_ and CS(h)-SeNPs to protect the GPx activities (Fig. 7a, c) and reduce LF accumulation (Fig. 7b, d) with the same Se dose (1 mg/kg, drug/body weight). The effects of the CS(l)-SeNPs could also be improved in a dose-dependent manner.

The dosages of the drugs were limited by their toxicity. The toxic doses of H_2_SeO_3_ and CS(l)-SeNPs were compared in Table 3. According to literature [51], the moderately toxic dose and highly toxic doses were categorized as 50–500 and 5–50 mg/kg (drug/body weight), respectively. The LD50 of H_2_SeO_3_ was 22.0 mg/kg with 95% confidence from 15.9 to 30.4 and was highly toxic while the CS(l)-SeNPs, with LD50 of 258.2 mg/kg with 95% confidence between 193.9 and 343.9, belonged to a moderately toxic substance. A similar result was reported by Wang et al. in which the LD50 of Se nanoparticle was at the level of 113.0 mg/kg with 95% confidence being 89.9–141.9 [6].

**Table 3:** Acute lethal effect of Se samples by oral administration

H_2_SeO_3_		CS(l)-SeNPs	
Selenium dose (mg drug/body)	Mouse mortality (%)	Selenium dose (mg drug/body)	Mouse mortality (%)
7.1	0	74.1	0
10.7	10	111.1	10
16.0	40	166.7	10
24.0	60	250.0	50
36.0	90	375.0	90

## Conclusions

In conclusion, the antioxidant abilities of the Se nanoparticles stabilized with different CS, i.e. CS(l)-SeNPs and CS(h)-SeNPs, could be enhanced by a 30-day storage process. The transdermal Se delivery efficiency of these CS-SeNPs was equivalent to that of selenite. The good abilities to penetrate cell or tissue have made these nanoparticles to be able to effectively inhibit ROS accumulation, reduce Se cytotoxicity, protect GPx activity and prevent LF accumulation, in vitro or in vivo. The UV-radiation or d-galactose tests indicated that the antioxidant capacities of CS-SeNPs were more evident in viscera than in skin of mice. However, regarding the aspect of dose effect control, CS(l)-SeNPs was found more efficient than CS(h)-SeNPs. From a more prospective point of view, we believe that further studies will be needed to explore the metabolic fate and long-term fate, stability and potential transformation of the chitosan selenium nanoparticles in vivo.

## Abbreviations

Se: selenium
Se^0^: the element Se with zero oxidation state
SeNPs: Se^0^ nanoparticles
CS: chitosan
Mws: molecular weigthts
CS-SeNPs: selenium nanoparticles stabilized with chitosan
CS(l)-SeNPs: selenium nanoparticles with the low molecular weight chitosan
CS(h)-SeNPs: selenium nanoparticles with the high molecular weight chitosan
ROS: reactive oxygen species
GPx: glutathione peroxidase
LF: lipofusin
H_2_SeO_3_: the seleninic acid
ABTS: 2, 2′-Azino-bis (3-ethylbenzothiazoline-6-sulfonic acid
DPPH: 2, 2-diphenyl-1-picrylhydrazyl
DAN: 2, 3-diaminonaphthalene
DCFH-DA: 2′,7′-dichlorofluorescein diacetate
DMP-30: 2,4,6-Tris(dimethylaminomethyl) phenol
DMEM: Dulbecco’s modified Eagle medium
NEAA: MEM nonessential amino acid solution
DMSO: dimethyl sulfoxide
PBS: potassium phosphate
CS3: chitosan with a molecular weight of less than 3 kDa
CS200: chitosan with a molecular weight of less than 200 kDa
K_2_S_2_O_8_: potassium persulfate
TCA: trichloroacetic acid
TBA: 2-thiobarbituric acid
STEM: scanning transmission electron microscopy
RSC%: the radicals scavenging activity
MDA: malondialdehyde
MTT: 3-(4, 5-dimethylthiazol-2-yl)-2, 5-diphenyltetrazolium bromide
AFS: hydride-generation atomic fluorescence spectrometry
ET-based: assays based on electron transfer
HAT-based: assays based on hydrogen atom transfer
